# Supplementary material for: Bacteria encode post-mortem protein catabolism that enables altruistic nutrient recycling
Source: Nat Commun. 2025 Feb 13;16:1400. doi: 10.1038/s41467-025-56761-6 (PMC11825663; doi:10.1038/s41467-025-56761-6)
Supplement: Supplementary file 1 — Supplementary Information [file 41467_2025_56761_MOESM1_ESM.pdf]

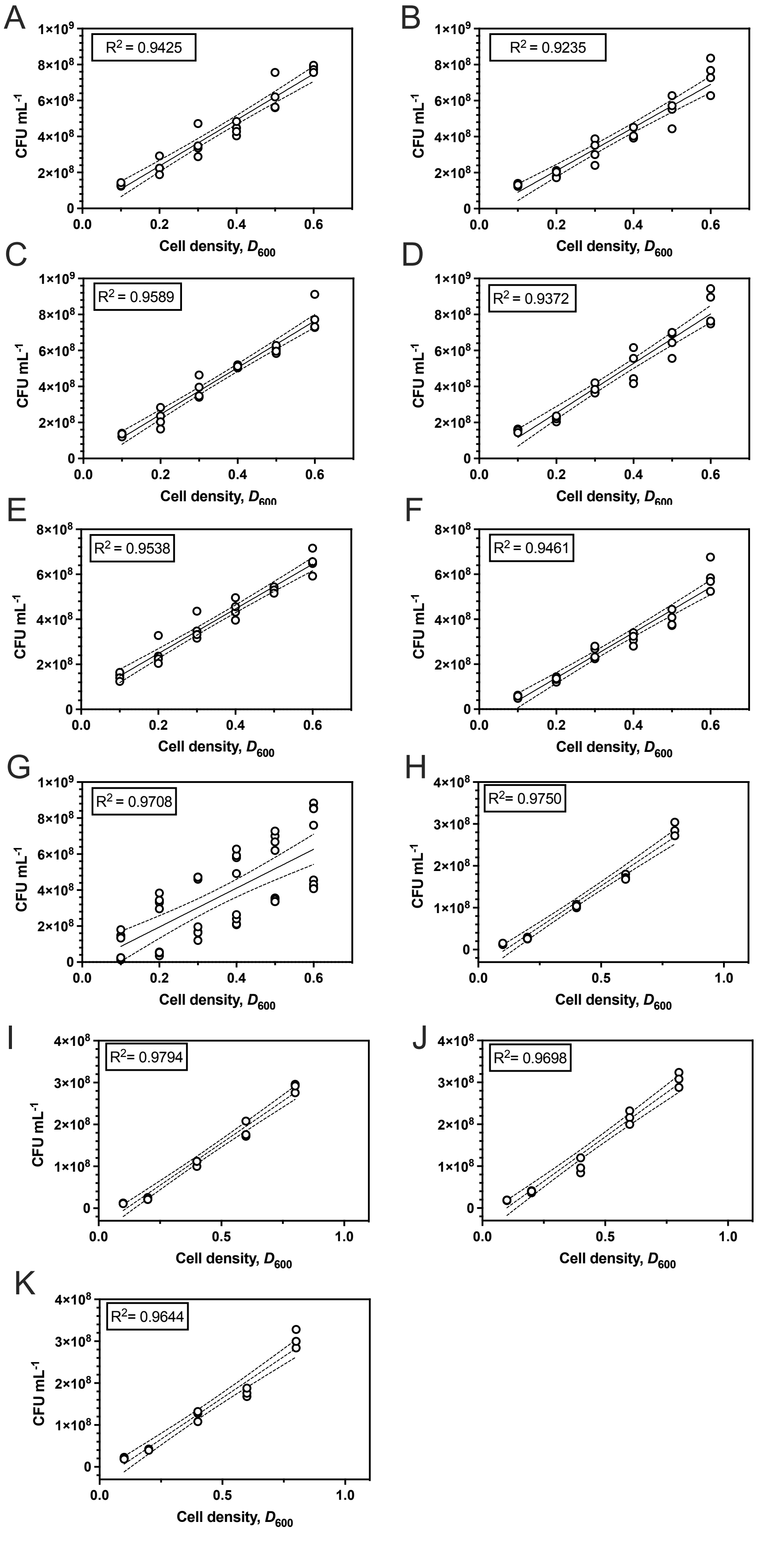

Figure S1. Plots of CFU mL^{-1} against cell density for all examined strains and lysates. R^2 value and best fit straight lines with ± 95% C.I. are provided in all plots **A.** WT cells with no added lysate (*n*=4). **B.** WT with Lon-null lysate (*n*=4). **C.** WT cells with WT lysate (*n*=4). **D.** WT cells with OmpT-null lysate (*n*=4). **E.** Lon-null cells with no added lysate (*n*=4). **F.** Lon-null cells with Lon-null lysate (*n*=4). **G.** Lon-null cells

with WT lysate (*n*=8). **H.** *B. subtilis* cells with no added lysate (*n*=4). **I.** *B. subtilis* cells with Lon-null lysate (*n*=4). **J.** *B. subtilis* cells with WT lysate (*n*=4). **K.** *B. subtilis* cells with *B. subtilis* lysate (*n*=4). Source data are provided as a Source Data file.

The slopes for panels **A-D** are not significantly different (p = 0.3577; F (DFn = 3, DFd = 88) = 1.09). This means that changes in cell density for *E. coli* BW25113 cells is correlated with an equivalent change in cell number regardless of which bacterial lysate is used in the experiment.

The slopes for panels **E-G** are not significantly different (p = 0.8582; F (DFn = 2, DFd = 90) = 0.1532). This means that changes in cell density for *E. coli* BW25113Δ*lon* cells is correlated with an equivalent change in cell number regardless of which bacterial lysate is used in the experiment.

The slopes for panels **H-K** are not significantly different (p = 0.4548; F (DFn = 3, DFd = 52) = 0.8854). This means that changes in cell density for *B. subtilis* cells is correlated with an equivalent change in cell number regardless of which bacterial lysate is used in the experiment.

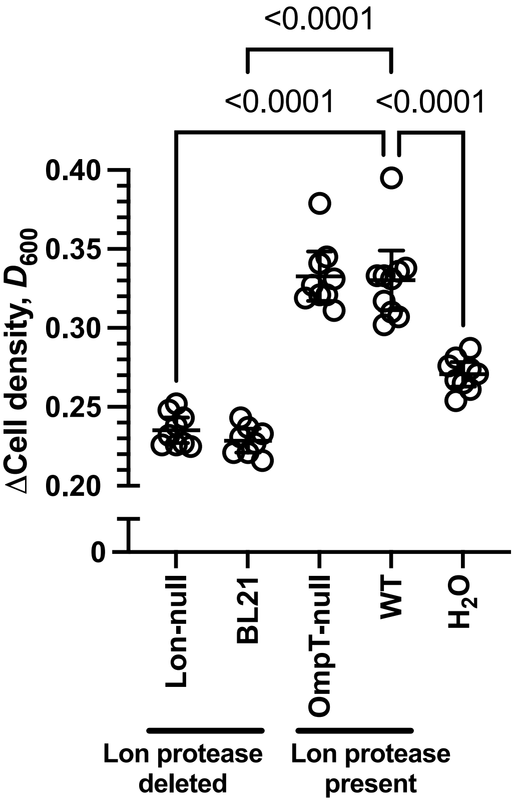

Figure S2. Lon protease derived from dead bacteria is required for nutrient recycling. Plots of the change in cell density of *E. coli* BW25113 grown at 37°C in M9/1% (v/v) glycerol media for 20 hrs in the presence or absence of lysate derived from the indicated *E. coli* strains. Mean ± 95% C.I., *p* values - one-way ANOVA with post hoc Tukey's multiple comparisons test, F (DFn = 4, DFd = 40) = 76.13. WT vs Lon-null*,* WT vs BL21, and WT vs H_2O $p < 0.0001$; WT vs OmpT-null $p = 0.9974$; H_2O vs Lon-null $p = 0.0008$. Lon-null, n=9; BL21, n=8; OmpT-null, n=9; WT, n=10; H_2O, n=9. Source data are provided as a Source Data file.

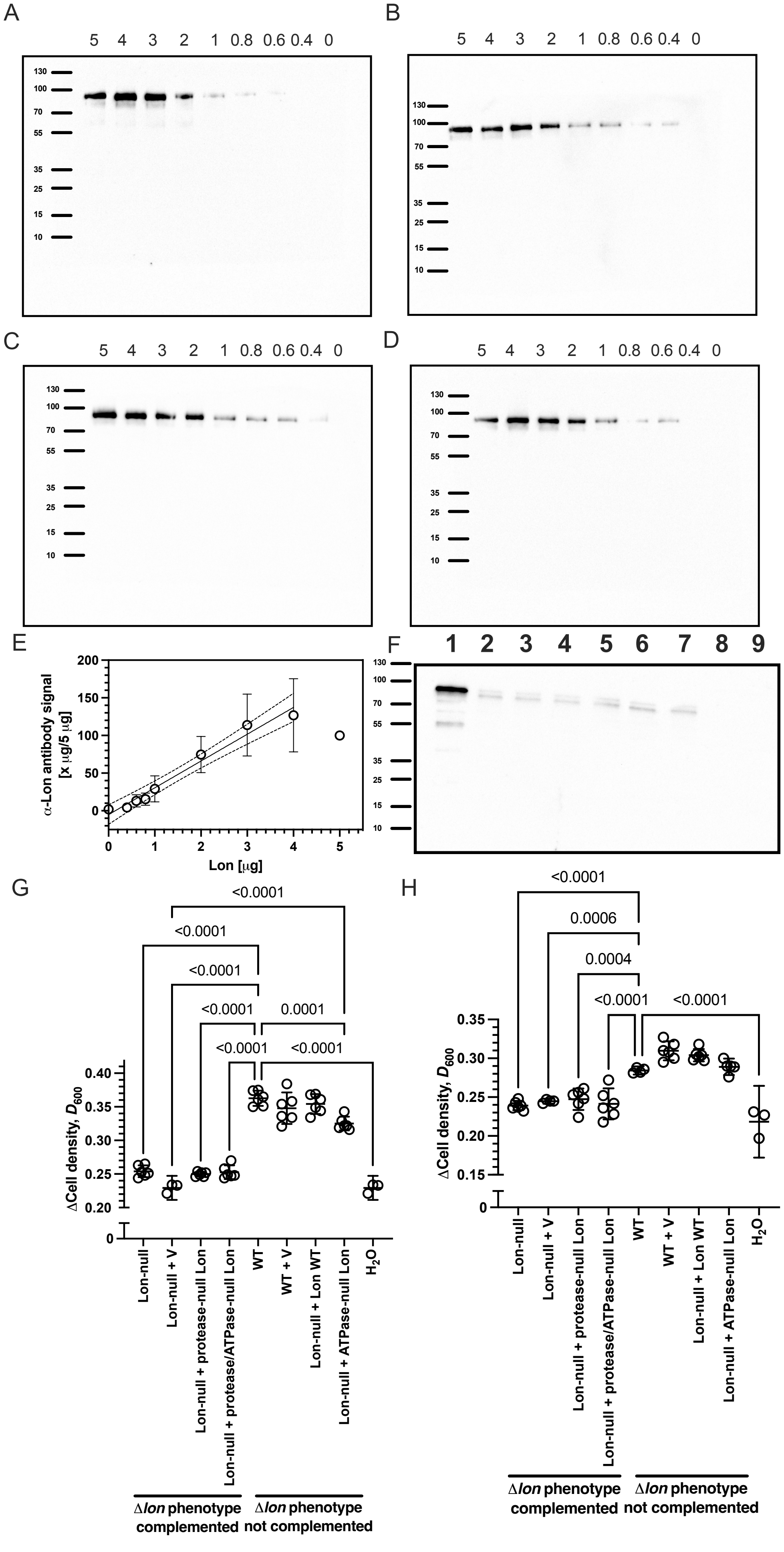
A
5 4 3 2 1 0.8 0.6 0.4 0
130
100
70
55
35
25
15
10
B
5 4 3 2 1 0.8 0.6 0.4 0
130
100
70
55
35
25
15
10
C
5 4 3 2 1 0.8 0.6 0.4 0
130
100
70
55
35
25
15
10
D
5 4 3 2 1 0.8 0.6 0.4 0
130
100
70
55
35
25
15
10
E
α-Lon antibody signal [x μg/5 μg]
200
150
100
50
0
0 1 2 3 4 5
Lon [μg]
F
1 2 3 4 5 6 7 8 9
130
100
70
55
35
25
15
10
G
<0.0001
<0.0001
<0.0001
<0.0001
0.0001
<0.0001
<0.0001
ΔCell density, D_{600}
0.40
0.35
0.30
0.25
0.20
0
Lon-null
Lon-null + V
Lon-null + protease-null Lon
Lon-null + protease/ATPase-null Lon
WT
WT + V
Lon-null + Lon WT
Lon-null + ATPase-null Lon
H_2O
Δ*lon* phenotype complemented
Δ*lon* phenotype not complemented
H
<0.0001
0.0006
0.0004
<0.0001
<0.0001
ΔCell density, D_{600}
0.35
0.30
0.25
0.20
0.15
0
Lon-null
Lon-null + V
Lon-null + protease-null Lon
Lon-null + protease/ATPase-null Lon
WT
WT + V
Lon-null + Lon WT
Lon-null + ATPase-null Lon
H_2O
Δ*lon* phenotype complemented
Δ*lon* phenotype not complemented

Figure S3. **A.** Western blot standard curve. Panels **A-D** show immunoblots for an α-Lon antibody signal with the indicated amount of loaded Lon recombinant protein [μg]. Panel **E** shows a plot of α-Lon antibody signal against the amount of loaded Lon protein. The α-Lon antibody signal values of the y-axis are normalised to the signal with 5 μg loaded Lon protein. The dotted line shows the 95% confidence interval for the linear regression. The linear regression slope is significantly non-zero ($p<0.0001$), and the plot was used to quantify *E. coli* endogenous Lon. **F.** Lon protein production in *E. coli* strains. Lon protein production was assessed by western blotting. Lane designations are **1.** 1 μg recombinant Lon protein. **2.** WT lysate. **3.** WT + pBAD33 lysate. **4.** Lon-null +pBAD33-Lon WT lysate. **5.** Lon-null +pBAD33-ATPase-null Lon lysate. **6.** Lon-null +pBAD33-protease-null Lon lysate. **7.** Lon-null +pBAD33-protease/ATPase-null Lon lysate. **8.** Lon-null lysate. **9.** Lon-null lysate +pBAD33. kDa indicates molecular weight markers. Individual blots represent independently produced panels of lysates. **G-H.** Plots of the change in cell density of *E. coli* BW25113 grown at 37°C in M9/1% (v/v) glycerol media for 20 hrs in the presence or absence of WT or Lon-null lysates and the indicated complementing empty (V) or Lon WT or mutant protein carrying plasmids. **F.** mean $\pm$ 95% C.I., *p* values - one-way ANOVA with post hoc Tukey's multiple comparisons test, F (DFn = 8, DFd = 39) = 111.9. WT vs Lon-null, WT vs Lon-null + V, WT vs Lon-null + protease-null Lon, WT vs Lon-null + protease/ATPase-null Lon, WT vs Lon-null + ATPase-null Lon, WT vs H_2O $p < 0.0001$; WT vs WT + V $p = 0.4650$, WT vs Lon-null + Lon WT $p = 0.9461$; H_2O vs Lon-null $p = 0.1199$. H_2O, Lon-null+V, n=3; WT, WT + V, Lon-null + Lon WT, Lon-null + ATPase-null Lon, Lon-null + protease-null Lon, Lon-null + protease/ATPase-null Lon, Lon-null, Lon-null + V, n=6. **G.** mean $\pm$ 95% C.I., *p* values - one-way ANOVA with post hoc Tukey's multiple comparisons test, F (DFn = 8, DFd = 37) = 40.96. WT vs Lon-null, WT vs Lon-null + protease/ATPase-null Lon, WT vs H_2O $p < 0.0001$; WT vs Lon-null + V $p = 0.0006$; WT vs WT + V $p = 0.0341$; WT vs Lon-null + Lon WT $p = 0.1905$; WT vs Lon-null + ATPase-null Lon $p = 0.9994$; WT vs Lon-null + protease-null Lon $p = 0.0004$; H_2O vs Lon-null $p = 0.2296$. H_2O, n=3; WT, Lon-null+V, n=4, Lon-null + ATPase-null Lon, n=5; WT, WT + V, Lon-null + Lon WT, Lon-null + protease-null Lon, Lon-null + protease/ATPase-null Lon, Lon-null, Lon-null + V, n=6. Source data are provided as a Source Data file.

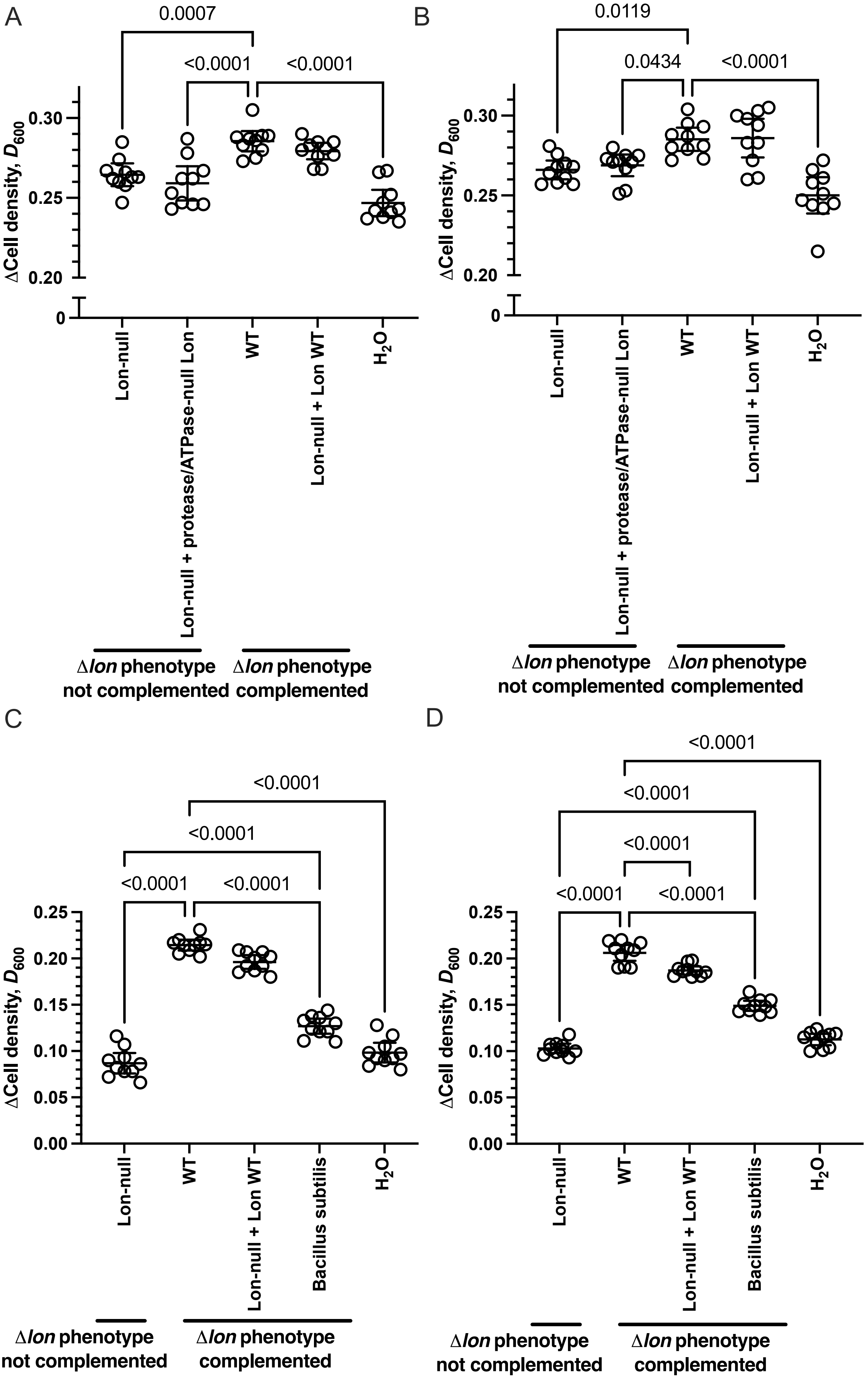

A
0.0007
<0.0001
<0.0001
ΔCell density, D_{600}
0.30
0.25
0.20
0
Lon-null
Lon-null + protease/ATPase-null Lon
WT
Lon-null + Lon WT
H_2O
Δ*lon* phenotype not complemented
Δ*lon* phenotype complemented
B
0.0119
0.0434
<0.0001
ΔCell density, D_{600}
0.30
0.25
0.20
0
Lon-null
Lon-null + protease/ATPase-null Lon
WT
Lon-null + Lon WT
H_2O
Δ*lon* phenotype not complemented
Δ*lon* phenotype complemented
C
<0.0001
<0.0001
<0.0001
<0.0001
ΔCell density, D_{600}
0.25
0.20
0.15
0.10
0.05
0.00
Lon-null
WT
Lon-null + Lon WT
Bacillus subtilis
H_2O
Δ*lon* phenotype not complemented
Δ*lon* phenotype complemented
D
<0.0001
<0.0001
<0.0001
<0.0001
<0.0001
ΔCell density, D_{600}
0.25
0.20
0.15
0.10
0.05
0.00
Lon-null
WT
Lon-null + Lon WT
Bacillus subtilis
H_2O
Δ*lon* phenotype not complemented
Δ*lon* phenotype complemented

Figure S4. Nutrient recycling is dependent on Lon protease activity. **A-B.** Plot of the change in cell density for Lon-null cells grown at 37°C in M9/1% (v/v) glycerol media for 20 hrs in the presence or absence of WT or Lon-null lysates and the indicated complementing empty plasmid (V;pBAD33) or Lon WT or mutant protein encoding plasmids (mean ± 95% C.I., *n*=10, *p* values - one-way ANOVA with post hoc Tukey's multiple comparisons test, **A.** F (DFn = 4, DFd = 45) = 20.71. WT vs H_2O, WT vs Lon-null + protease/ATPase-null Lon $p < 0.0001$; WT vs Lon-null $p = 0.0007$; H_2O vs Lon-null + Lon WT $p < 0.0001$. **B.** F (DFn = 4, DFd = 45) = 13.92). WT vs H_2O $p < 0.0001$; WT vs Lon-null $p = 0.0119$; WT vs Lon-null + protease/ATPase-null Lon $p = 0.0434$; H_2O vs Lon-null + Lon WT $p < 0.0001$. **C-D.** Plots of the change in cell density for *Bacillus subtilis* grown at 30°C in M9/1% (v/v) glucose media for 20 hrs in the presence or absence of WT, Lon-null or *B. subtilis* lysates and the indicated Lon WT protein-encoding plasmid (mean ± 95% C.I., *n*=10, *p* values - one-way ANOVA with post hoc Tukey's multiple comparisons test, **C.** F (DFn = 4, DFd = 45) = 219.9) WT vs Lon-null, *Bacillus subtilis*, WT vs H_2O $p < 0.0001$; H_2O vs Lon-null + Lon WT $p < 0.0001$. **D** F (DFn = 4, DFd = 45) = 280.8). WT vs Lon-null, WT vs *Bacillus subtilis*, WT vs H_2O $p < 0.0001$; H_2O vs Lon-null + Lon WT $p < 0.0001$. Source data are provided as a Source Data file.

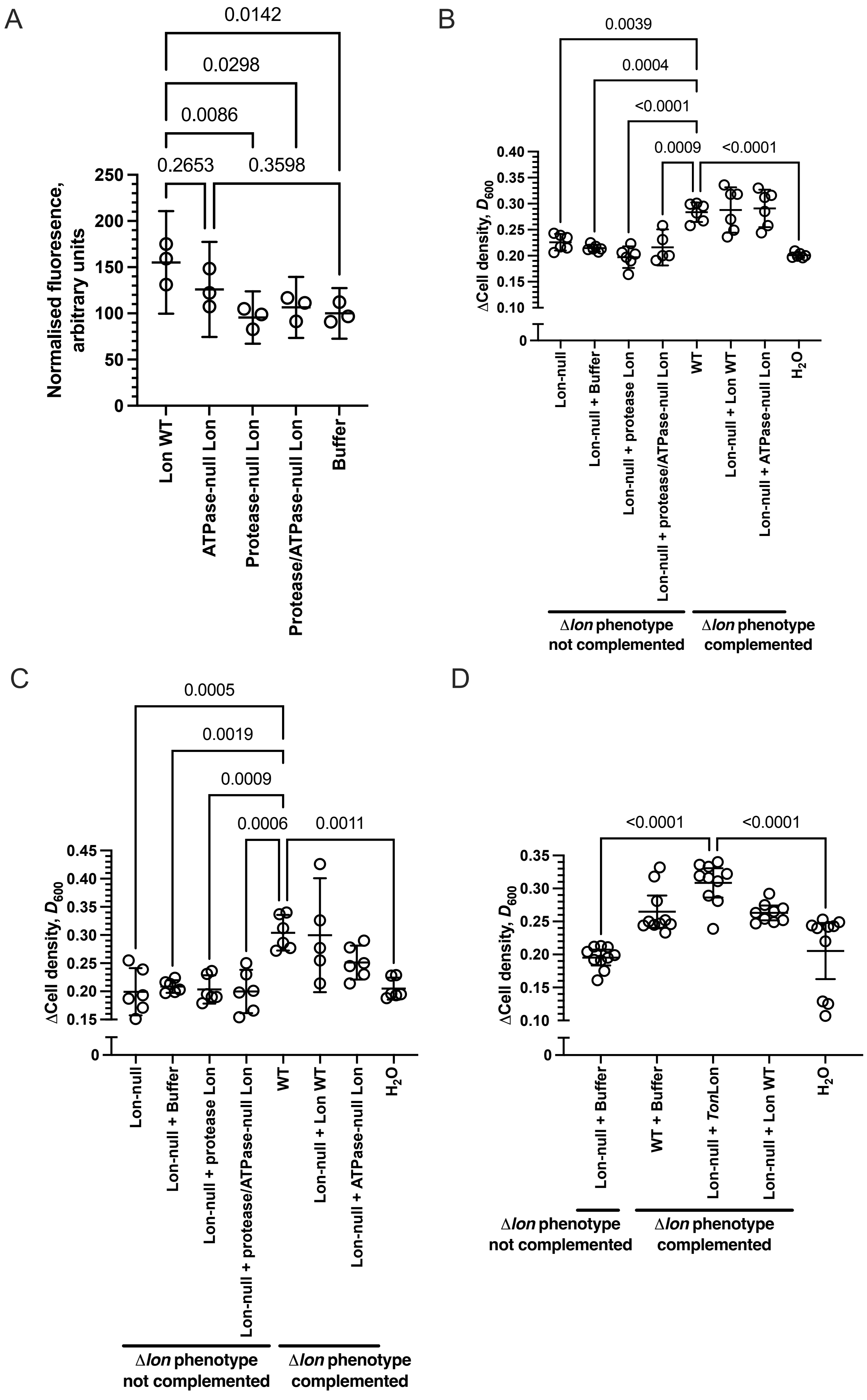
A
0.0142
0.0298
0.0086
0.2653
0.3598
Normalised fluoresence, arbitrary units
250
200
150
100
50
0
Lon WT
ATPase-null Lon
Protease-null Lon
Protease/ATPase-null Lon
Buffer
B
0.0039
0.0004
<0.0001
0.0009
<0.0001
ΔCell density, D_{600}
0.40
0.35
0.30
0.25
0.20
0.15
0.10
0
Lon-null
Lon-null + Buffer
Lon-null + protease Lon
Lon-null + protease/ATPase-null Lon
WT
Lon-null + Lon WT
Lon-null + ATPase-null Lon
H_2O
Δ*lon* phenotype not complemented
Δ*lon* phenotype complemented
C
0.0005
0.0019
0.0009
0.0006
0.0011
ΔCell density, D_{600}
0.45
0.40
0.35
0.30
0.25
0.20
0.15
0
Lon-null
Lon-null + Buffer
Lon-null + protease Lon
Lon-null + protease/ATPase-null Lon
WT
Lon-null + Lon WT
Lon-null + ATPase-null Lon
H_2O
Δ*lon* phenotype not complemented
Δ*lon* phenotype complemented
D
<0.0001
<0.0001
ΔCell density, D_{600}
0.35
0.30
0.25
0.20
0.15
0.10
0
Lon-null + Buffer
WT + Buffer
Lon-null + *Ton*Lon
Lon-null + Lon WT
H_2O
Δ*lon* phenotype not complemented
Δ*lon* phenotype complemented

Figure S5. Nutrient recycling depends on post-mortem Lon protease activity. **A.** Analysis of ATP-independent Lon protease activity. A plot of FITC fluorescence at 525 nm after FITC-casein degradation with the indicated Lon proteins and buffer control (mean ± 95% C.I., *n*=3, *p* values - one-way ANOVA with post hoc Tukey's multiple comparisons test, F (DFn = 4, DFd = 10) = 6.644). Lon WT vs Protease-null Lon *p* = 0.0086; Lon WT vs Protease/ATPase-null Lon *p* = 0.0298; Lon WT vs Buffer *p* = 0.0142; Lon WT vs ATPase-null Lon *p* = 0.2653; ATPase-null Lon vs Buffer *p* = 0.3598. **B-D.** Plots of the change in cell density for WT cells grown at 37°C in M9/1% (v/v) glycerol media for 20 hrs in the presence or absence of WT or Lon-null lysates and the indicated recombinant Lon proteins. **B.** Wild type and mutant *E. coli* Lon protease (mean ± 95% C.I., panel B, *n*=6; panel C, *n*=6; panel D, *n*=10, *p* values - one-way ANOVA with post hoc Tukey's multiple comparisons test, F (DFn = 7, DFd = 39) = 16.90). WT vs Lon-null *p* = 0.0039; WT vs Lon-null + buffer *p* = 0.0004, WT vs Lon-null + protease-null Lon, WT vs H_2O *p* < 0.0001; WT vs Lon-null + protease/ATPase-null Lon *p* = 0.0009; WT vs Lon-null + ATPase-null Lon *p* = 0.9995; H_2O vs Lon-null *p* = 0.6267. **C.** Wild type and mutant *E. coli* Lon protease (mean ± 95% C.I., *p* values - one-way ANOVA with post hoc Tukey's multiple comparisons test, F (DFn = 7, DFd = 39) = 8.247). WT vs Lon-null *p* = 0.0005; WT vs Lon-null + buffer *p* = 0.0019, WT vs Lon-null + protease-null Lon *p* = 0.0009, WT vs H_2O *p* = 0.0011; WT vs Lon-null + protease/ATPase-null Lon *p* = 0.0006; WT vs Lon-null + ATPase-null Lon *p* = 0.2523; H_2O vs Lon-null *p* > 0.9999. **D.** Wild type *E. coli* Lon protease and *Thermococcus onnurineus* NA1 Lon protease (*Ton*Lon) (mean ± 95% C.I., *p* values - one-way ANOVA with post hoc Tukey's multiple comparisons test, F (DFn = 4, DFd = 45) = 17.89). H_2O vs WT + buffer *p* = 0.0036; H_2O vs Lon-null + *Ton*Lon *p* < 0.0001, WT vs Lon-null + Lon WT *p* = 0.0043; H_2O vs Lon-null + buffer *p* = 0.9703. Source data are provided as a Source Data file.

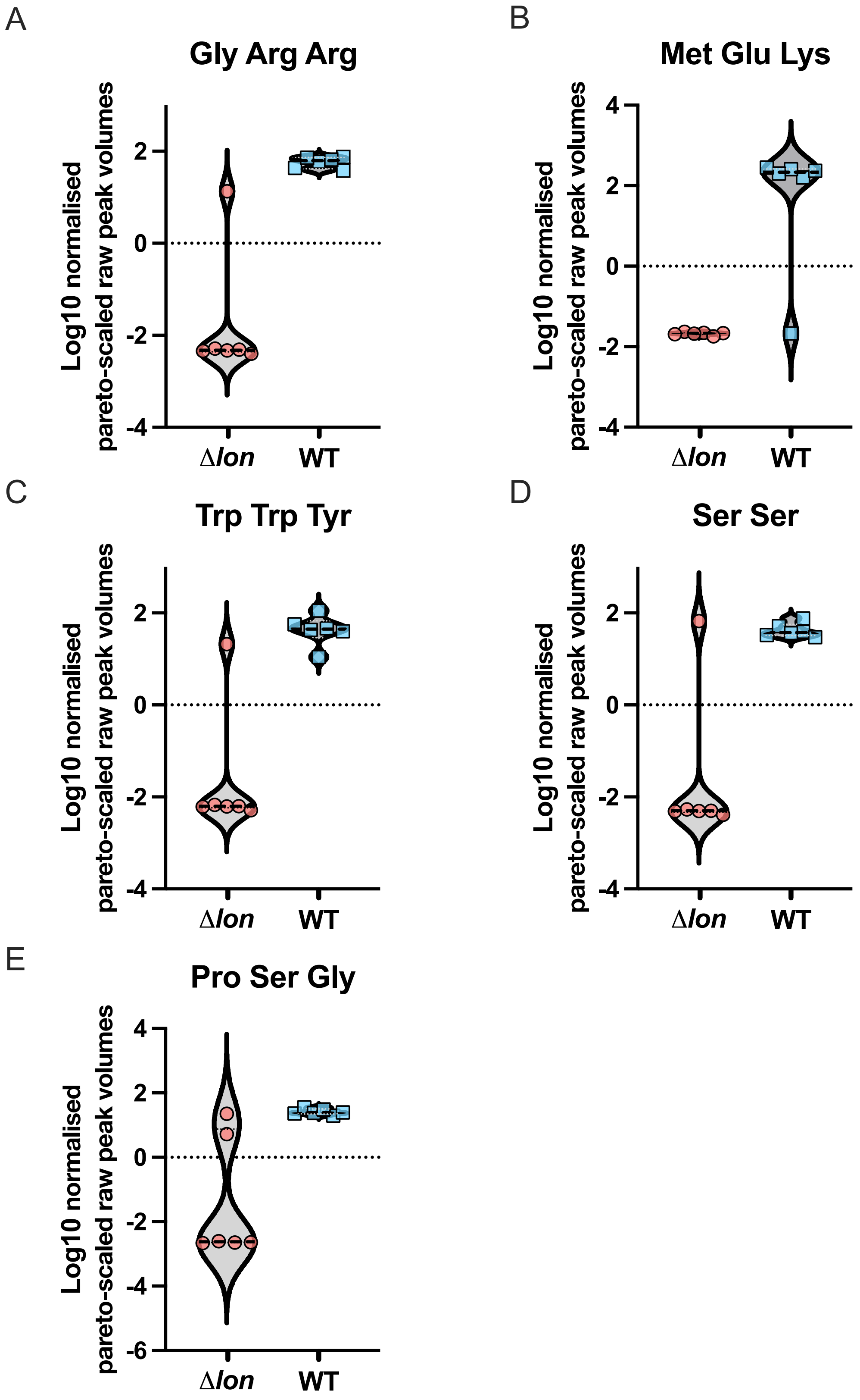

Figure S6. Lon protease is required for small peptide production in *E. coli* lysate. Violin plots (*n*=6) of the top 5 peptides of Figure 4 are shown for WT and Lon-null post-mortem bacterial lysates. Source data are provided as a Source Data file.
