# Supplementary material for: Bacteria encode post-mortem protein catabolism that enables altruistic nutrient recycling
Source: Nat Commun. 2025 Feb 13;16:1400. doi: 10.1038/s41467-025-56761-6 (PMC11825663; doi:10.1038/s41467-025-56761-6)
Supplement: Supplementary file 2 — Reporting Summary [file 41467_2025_56761_MOESM2_ESM.pdf]

nature portfolio

Corresponding author(s): Martin Cann
Last updated by author(s): Jan 15, 2025

Reporting Summary

Nature Portfolio wishes to improve the reproducibility of the work that we publish. This form provides structure for consistency and transparency in reporting. For further information on Nature Portfolio policies, see our Editorial Policies and the Editorial Policy Checklist.

Statistics

For all statistical analyses, confirm that the following items are present in the figure legend, table legend, main text, or Methods section.

n/a	Confirmed	
☐	☒	The exact sample size (*n*) for each experimental group/condition, given as a discrete number and unit of measurement
☐	☒	A statement on whether measurements were taken from distinct samples or whether the same sample was measured repeatedly
☐	☒	The statistical test(s) used AND whether they are one- or two-sided<br>*Only common tests should be described solely by name; describe more complex techniques in the Methods section.*
☒	☐	A description of all covariates tested
☐	☒	A description of any assumptions or corrections, such as tests of normality and adjustment for multiple comparisons
☐	☒	A full description of the statistical parameters including central tendency (e.g. means) or other basic estimates (e.g. regression coefficient) AND variation (e.g. standard deviation) or associated estimates of uncertainty (e.g. confidence intervals)
☐	☒	For null hypothesis testing, the test statistic (e.g. *F*, *t*, *r*) with confidence intervals, effect sizes, degrees of freedom and *P* value noted<br>*Give P values as exact values whenever suitable.*
☒	☐	For Bayesian analysis, information on the choice of priors and Markov chain Monte Carlo settings
☒	☐	For hierarchical and complex designs, identification of the appropriate level for tests and full reporting of outcomes
☒	☐	Estimates of effect sizes (e.g. Cohen's *d*, Pearson's *r*), indicating how they were calculated

Our web collection on statistics for biologists contains articles on many of the points above.

Software and code

Policy information about availability of computer code

Data collection	N/A
Data analysis	GraphPad Prism v 10.4.1, MassHunter Data Acquisition v10.0, PNNL PreProcessor v2020.03.23, MassHunter IM-MS Browser v 10.0, Mass Profiler v 10.0, MetaboAnalyst 6.0

For manuscripts utilizing custom algorithms or software that are central to the research but not yet described in published literature, software must be made available to editors and reviewers. We strongly encourage code deposition in a community repository (e.g. GitHub). See the Nature Portfolio guidelines for submitting code & software for further information.

Data

Policy information about availability of data

All manuscripts must include a data availability statement. This statement should provide the following information, where applicable:

- Accession codes, unique identifiers, or web links for publicly available datasets
- A description of any restrictions on data availability
- For clinical datasets or third party data, please ensure that the statement adheres to our policy

Source data are provided with this paper. Peptide analysis data is availability through Metabolights study number MTBLS10044.

Research involving human participants, their data, or biological material

Policy information about studies with human participants or human data. See also policy information about sex, gender (identity/presentation), and sexual orientation and race, ethnicity and racism.

Reporting on sex and gender	N/A
Reporting on race, ethnicity, or other socially relevant groupings	N/A
Population characteristics	N/A
Recruitment	N/A
Ethics oversight	N/A

Note that full information on the approval of the study protocol must also be provided in the manuscript.

Field-specific reporting

Please select the one below that is the best fit for your research. If you are not sure, read the appropriate sections before making your selection.

☒ Life sciences ☐ Behavioural & social sciences ☐ Ecological, evolutionary & environmental sciences

For a reference copy of the document with all sections, see nature.com/documents/nr-reporting-summary-flat.pdf

Life sciences study design

All studies must disclose on these points even when the disclosure is negative.

Sample size	ample sizes were determined as a count of individual experimental replicates and were selected as compatible with the statistical tests used and for sufficient power. Power calculations are detailed in the manuscript where relevant.
Data exclusions	No data was excluded
Replication	All experiments represent replicate experiments performed independently . All replicates were included and n is indicated in legends
Randomization	All findings were independent of the order of performance of experimental replicates
Blinding	Blinding not relevant as a single experimentalist establishes the experiments and analyses the data

Reporting for specific materials, systems and methods

We require information from authors about some types of materials, experimental systems and methods used in many studies. Here, indicate whether each material, system or method listed is relevant to your study. If you are not sure if a list item applies to your research, read the appropriate section before selecting a response.

Materials & experimental systems

n/a	Involved in the study	
☐	☒	Antibodies
☒	☐	Eukaryotic cell lines
☒	☐	Palaeontology and archaeology
☒	☐	Animals and other organisms
☒	☐	Clinical data
☒	☐	Dual use research of concern
☒	☐	Plants

Methods

n/a	Involved in the study	
☒	☐	ChIP-seq
☒	☐	Flow cytometry
☒	☐	MRI-based neuroimaging

Antibodies

Antibodies used	anti-Lon protease primary antibody (Sino Biological, 40219-T24). goat anti-rabbit secondary antibody (Abcam, ab205718)

Validation	anti-Lon protease primary antibody validated for use in western blotting by the authors. Reactivity confirmed by control experiments included in blots in Figure S3A-D using purified recombinant Lon protease. goat anti-rabbit secondary antibody validated for use in western blotting by abcam. All antibody details provided at https://www.abcam.com/en-us/products/secondary-antibodies/goat-rabbit-igg-h-l-hrp-ab205718?srsltid=AfmBOoqYlgKq1G-aERn6TBw36A8uDrGRQ2SngJk-jBgGWywu5p3Q46bX

Plants

Seed stocks	N/A
Novel plant genotypes	N/A
Authentication	N/A
